# New dimensions and new tools to realize the potential of RDoC: digital phenotyping via smartphones and connected devices

**DOI:** 10.1038/tp.2017.25

**Published:** 2017-03-07

**Authors:** J Torous, J-P Onnela, M Keshavan

**Affiliations:** 1 Department of Psychiatry, Beth Israel Deaconess Medical Center, Harvard Medical School, Boston, MA, USA; 2 Division of Clinical Informatics, Beth Israel Deaconess Medical Center, Harvard Medical School, Boston, MA, USA; 3 Department of Biostatistics, Harvard T.H. Chan School of Public Health, Boston, MA, USA

## Abstract

Mobile and connected devices like smartphones and wearable sensors can facilitate the collection of novel naturalistic and longitudinal data relevant to psychiatry at both the personal and population level. The National Institute of Mental Health's Research Domain Criteria framework offers a useful roadmap to organize, guide and lead new digital phenotyping data towards research discoveries and clinical advances.

## Brief overview of NIMH's RDoC

The classification of psychiatric disorders remains unsatisfactory. Although reliability of diagnoses has improved with successive editions of the Diagnostic and Statistical Manual of Mental Disorders,^1^ the validity of diagnostic categories remains in question.^2^ As an alternative to symptom-based classifications, the National Institute of Mental Health's Research Domain Criteria (NIMH's RDoC) was launched in 2011 as an effort to facilitate precision medicine in psychiatry and offer a novel framework to organize and understand research knowledge.^3^ RDoC seeks to quantify illnesses beyond patient self-report and instead considers them through of a series of constructs (concepts representing a specified functional dimension of behavior, for example, acute threat or potential threat) measured across a spectrum of units of analysis ranging from genes to behaviors.^4, 5^ By focusing on individual constructs, for example, potential threat instead of broader anxiety, and including measurements of biological units at level of genes, molecules, cells and neural circuits, as well as a range of observable units of analysis such as physiology, behavior and self-report, NIMH's RDoC proposes that we may be able to better define and understand the mechanism of mental illness. RDoC is not simply a data collection matrix with rows representing constructs and columns representing units of analysis; it is designed as a framework to organize data such that knowledge gaps can be identified and hypothesis-driven research can move forward connecting translational domains at cell, molecular, circuit and behavioral levels. (The RDoC Matrix can be directly accessed here: https://www.nimh.nih.gov/research-priorities/rdoc/constructs/rdoc-matrix.shtml.) However, to date, linking biologically based psychiatric research to clinically observable symptoms has proven challenging.^6, 7^

## Need for new tools and data

This challenge to realize a unified biological model of psychiatric illness is not new,^8, 9^ and may reflect that NIMH's RDoC reconceptualization of illness, like others before it, is alone not sufficient. Rather, innovative models such as NIMH's RDoC need new tools and new data streams to inform the framework much like the microbiology needed its new tools for data gathering in the form of the microscope. With the increasing recognition that what we today label as psychiatric illness likely represents only one end of a continuous spectrum,^10^ there is a need to better understand less overt manifestations of illness. Considering psychosis as a trans-diagnostic and extended phenotype in the general population,^11^ what do the NIMH's RDoC constructs such as negative and positive valence systems, cognitive systems, social process, and arousal and regulatory systems look like in those with transitory and subthreshold psychotic experiences, as compared to those with clinically manifest psychotic disorders? Capturing and quantifying such patient experiences traditionally would be difficult, especially as many patients may only experience brief or transitory symptoms that are often missed in traditional infrequent assessments.^12^

## Digital phenotyping

**Figure 1:**
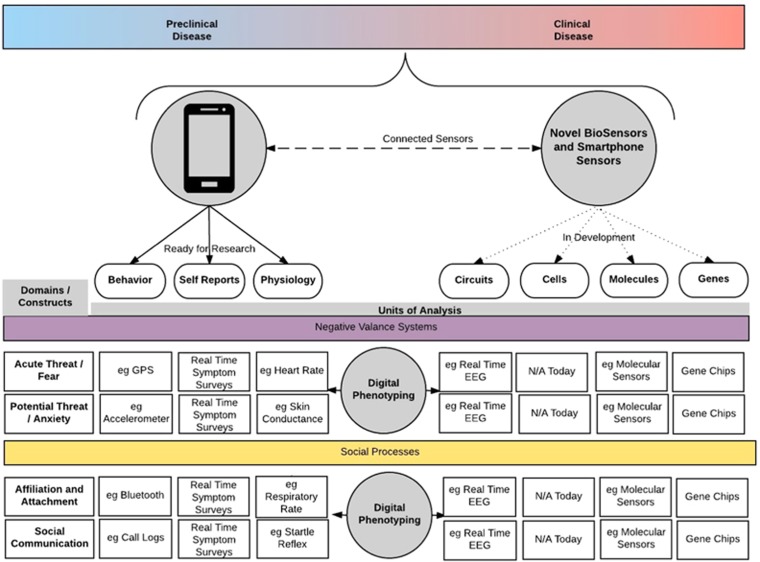
Although smartphone data may already help identify symptoms in those with recognized illness (colored red), the potential of using an RDoC framework extends to the general population with preclinical as well as non-observable symptoms (colored blue), smartphones will enable large-scale RDoC-guided data collection that may reveal trans-diagnostic extended phenotypes. This figure only shows a subset of the RDoC matrix, which includes further constructs not displayed. EEG, electroencephalogram; RDoC, Research Domain Criteria.

Connected devices such as smartphones, wearable devices and tablets now offer novel tools to capture several units of analysis of the NIMH's RDoC framework, making it possible to collect new data streams that were previously either difficult or nearly impossible to capture. Through offering real-time self-assessment surveys on the phone, it is now possible to collect time-stamped symptom surveys in a more convenient and less burdensome manner than using prior ecological momentary assessment methods.^12, 13^ Utilizing phone sensors, such as GPS, to determine temporal mobility patterns, voice recordings to detect speech and vocal markers, and call and text logs to indicate degrees of social interaction, the phone is now able to offer objective measures of behavior in a less burdensome manner than prior actigraphy efforts.^14^ Finally, with many smartphone and wearable sensors now able to detect pulse, galvanic skin conductance, temperature and ambient light among other capabilities, collecting real-time physiological data from subjects is practical for psychiatric research.^15, 16^ Considering Figure 1 below, the self-report, behavior and physiology units of analysis from RDoC can now be captured from many commercially available smartphones, biosensors and other connected devices. This moment-by-moment quantification of the individual-level human phenotype *in situ* using data from smartphones and other personal digital devices can be considered digital phenotyping.^14^ Although the ability to record real-time self-reported symptoms, objective measures of behavior and continuous physiology from millions of individuals was not the focus of NIMH's RDoC original conceptualization, the rapid recent expansion of smartphones and connected devices offers both a useful new tool and data streams. As portable biosensors and smartphones advance, in the near future we may soon be able to collect biological units of analysis such as genetic, molecular and electroencephalogram data in a reproducible and transparent manner.^17, 18, 19^ In practical terms, however, the use of digital devices to collect such data may be currently limited and most practical for behavior, self-reports and physiology.

## NIMH's RDoC and digital phenotyping

Although traditional research efforts studying genes, molecules, cells and neural circuits have struggled to find reproducible clinical correlates,^20^ the field's prior difficulties in quantifying subjects' physiology, behavior and self-reported symptoms may explain why this has been a challenge. Assuming that many observable phenotypes of psychiatric illnesses are temporally dynamic, environmentally influenced and socially modulated—reliably capturing such measures without tools such as smartphones is difficult. Further, with many psychiatric illnesses impacting cognitive functioning, relying on subject self-report for data on symptoms, behavior and even physiology may be unreliable. Instead, approaching the problem with smartphone-based RDoC-like data offers a plethora of quantified information collected not only at periodic study visits but also in the subject's real-world environment, in real time, possibly over long time periods, with minimal recall bias. Digital phenotyping may offer a new lens through which the field's genetic, molecular, cellular and neural circuitry data better correlate with clinically observable psychopathology, thus elucidating translational mechanisms of illness.^14, 21^ Consider a patient with clinically diagnosed agoraphobia and panic disorder. Using the construct of acute threat or fear, a smartphone in such a patient could collect self-reported data through diagnostic and assessment scales delivered as surveys on the phone, behavioral data such as avoidant behavior through GPS, facial expressions through the camera, errors/response times through cognitive assessments delivered as tap tests, and physiological data such as heart rate and skin conductance. The location of avoidance based on GPS data could indicate the source of the threat (for example, grocery store or a public eating place). Such observations can offer a great deal more information than is possible simply by periodic clinical assessments. Observations of such episodes during sleep may raise the question of whether another illness such as temporal lobe epilepsy may underlie the panic attacks. Such data, in individuals not yet diagnosed with an illness can potentially help identify preclinical indications of risk for the disorder. As another example, the construct of affiliation of attachment, which was previously difficult to quantify and measure proximity preferences of individuals. Now using bluetooth sensing on smartphones, which can detect proximity to similar sensors on others' smartphones, it is feasible to monitor social patterns in an unobtrusive manner that can provide data on how individuals interact with others. Utilizing tools such as the Beiwe platform that is a research grade smartphone app collecting digital phenotyping data with a supporting analytical toolset,^14^ clinical studies can now longitudinally collect these data streams on subjects' own personal smartphones. Although the ability to capture such data does not inherently mean that it is clinically valid or even useful, it offers new opportunities for clinical studies to field-test and validate these approaches.

If NIMH's RDoC offers a roadmap to conceptualize psychiatric illness in a new light, smartphones and digital phenotyping offer added fuel to power new discoveries along this guided route. Unlike prior actigraphy research efforts that were hampered by inconvenient technology and high study costs, the high rates of smartphone ownership mean that large population level studies of psychiatric illness can be conducted at relatively low cost as has been recently demonstrated in major depressive disorder.^22^ It is possible to imagine how the ubiquity of smartphones will enable studies to capture rare digital phenotypes from the general population, as well as ones we today do not yet recognize; digital phenotyping may also identify which subjects to conduct more extensive genetic, molecular, cellular and neuroimaging investigations on. It is also possible to delineate the trans-diagnostic, and translational signatures of psychopathological dimensions at a preclinical level in the community and map that on to what we see in clinical populations. Such large-scale studies will be necessary to capture and quantify extended phenotypes of illness, but with the RDoC framework and population already owning^23^ and willing to use smartphones for clinical research,^24, 25^ we already have the roadmap and tools available today.

## Concerns

The scope and scale of digital phenotyping efforts also raise considerable barriers. Privacy and confidentiality issues must be fully addressed before digital tools can be broadly applied. Likewise, transparency in data processing and analysis is needed to build trust and ensure reproducibility. The ethics associated with these digital tools require a more collaborative dialog between all stakeholders and greater subject and patient involvement. Although NIMH's RDoC may offer a way forward for digital phenotyping, the answer to the issues is less straightforward and a current struggle for many fields of healthcare even outside of psychiatry. New collaborations with data science fields, increased education about digital tools for both patients and clinicians, and emphasizing digital privacy first are all important first steps.

## Conclusion

Combining the RDoC framework with digital phenotyping offered from smartphones and other connected devices presents a unique opportunity for psychiatric research. Through incorporating the potential of these new digital technologies into RDoC framed clinical questions, psychiatry can now explore new dimensions of pathology largely inaccessible only a few years before.
